# Reprogramming of Mice Primary Hepatocytes into Insulin-Producing Cells by Transfection with Multicistronic Vectors

**DOI:** 10.1155/2014/716163

**Published:** 2014-05-19

**Authors:** Haizhao Luo, Rongping Chen, Rui Yang, Yan Liu, Youping Chen, Yi Shu, Hong Chen

**Affiliations:** ^1^Department of Endocrinology, Zhujiang Hospital, Southern Medical University, No. 253 Gong Ye Road, Guangzhou 510282, China; ^2^Department of Endocrinology, Nanhai Hospital, Southern Medical University, No. 40 Foping Road, Foshan 528200, China

## Abstract

The neogenesis of insulin-producing cells (IPCs) from non-beta-cells has emerged as a potential method for treating diabetes mellitus (DM). Many groups have documented that activation of pancreatic transcription factor(s) in hepatocytes can improve the hyperglycemia in diabetic mice. In the present study, we explored a novel protocol that reprogrammed primary hepatocytes into functional IPCs by using multicistronic vectors carrying pancreatic and duodenal homeobox-1 (Pdx1), neurogenin 3 (Ngn3), and v-musculoaponeurotic fibrosarcoma oncogene homolog A (MafA). These triple-transfected cells activated multiple beta-cell genes, synthesized and stored considerable amounts of insulin, and released the hormone in a glucose-regulated manner in vitro. Furthermore, when transplanted into streptozotocin-induced diabetic mice, the cells markedly ameliorated glucose tolerance. Our results indicated that ectopic expression of Pdx1, Ngn3, and MafA facilitated hepatocytes-to-IPCs reprogramming. This approach may offer opportunities for treatment of DM.

## 1. Introduction

According to data from International Diabetes Federation (IDF), there were about 371 million people suffering from diabetes mellitus (DM) in 2012 [1]. Islet transplantation has been considered as a promising strategy for curing DM, whereas it is limited by both scarcity of donor cells and immunologic rejection. It is widely believed that cell replacement for curing DM will be applied on a large scale only when new sources of insulin-producing cells (IPCs) are discovered.

On the phase of organogenesis, the same as pancreatic islet beta-cells, hepatocytes also are derived from endoderm and both of them share many of their epigenomes, such as glucose transporter-2 and glucokinase (Gk) [2]. Conversion between hepatocytes and pancreatic islet beta-cells may therefore require fewer epigenetic changes. Hepatocytes can serve as the potential source of IPCs.

Transcription factors are the elements that regulate differentiation and development of cells. It was documented that hepatocytes could be reprogrammed into IPCs by introducing certain transcription factor(s). Pancreatic and duodenal homeobox-1 (Pdx1) was proved to play a crucial role on pancreatic morphogenesis and function in postnatal islet [3]. Ferber et al. reported that ectopic expression of Pdx1 could induce the conversion from hepatocytes to IPCs and improve the hyperglycemia in streptozotocin- (STZ-) treated diabetic mouse [4]. Thereafter, several studies have confirmed that many other factors involved in pancreas development, including neurogenin 3 (Ngn3) [5], betacellulin [6], neurogenic differentiation (NeuroD) [7], and v-musculoaponeurotic fibrosarcoma oncogene homolog A (MafA) [8], could also turn on endocrine program in hepatocytes but not to generate functional beta-cells. Recently, an exciting and interesting breakthrough in islet regeneration was found by Melton and his colleagues. Among more than 1100 pancreas-associated transcription factors, they established a specific combination (Pdx1, Ngn3, and MafA) which was a most efficient precept in reprogramming nonpancreatic beta-cells into IPCs that closely resemble endogenous *β*-cells [9].

Many groups have attempted to set up a practical induction of islet regeneration by virus-mediated protocol. However, safety concerns have been the main bottleneck to the studies of viral gene delivery. In 1999, the death of a volunteer due to gene therapy in a clinical trial was caused by administering adenovirus vectors within 98 hours [10]. The autopsy report revealed that the patient succumbed to multiorgan failure owing to the fatal immune response triggered by the administered adenovirus [11]. In order to allow clinical use of reprogramming, it is urgent to explore a feasible nonviral strategy.

Here, we showed that coexpression of Pdx1, Ngn3, and MafA in primary hepatocytes induced hepatocytes-to-IPCs reprogramming and reversal of hyperglycemia in diabetic animals by using multicistronic vectors via liposome.

## 2. Materials and Methods

### 2.1. Plasmid Construction

The transcription factors of mouse Pdx1, Ngn3, and MafA (gene ID: 008814.3, 009719.6, 194350.1) were PCR-amplified from total RNA and ligated with an open reading frame (ORF) or 3′-untranslated regions (UTR), subsequently, cloned into a shuttle vector pcDNA3.1 (+) (Clontech, USA), respectively. Electrophoretic analysis and gene sequencing were conducted to make sure that all the recombinant plasmids were correct.

### 2.2. Cell Culture

4-week-old male C57BL/6J mice (Laboratory Animal Center of Southern Medical University, Guangzhou, China) were kept in a controlled-temperature (22–25°C) animal room, with a 12 h light and 12 h dark cycle. Pelleted commercial chow (Laboratory Animal Center of Southern Medical University) and purified water were available. The experimental procedures performed in this study were in accordance with the guidelines of the Institutional Animal Ethics Committee for the Care and Use of Laboratory Animals. Hepatocytes were isolated and purified from the animals by in situ collagenase perfusion, as mentioned previously [12]. The hepatocytes were plated at 6-well plates precoated with collagen (Corning, USA) and incubated at 37°C with 5% humidified CO_2_.

### 2.3. Transfection

Primary hepatocytes were transfected by Lipofectamine 2000 (Invitrogen, USA) according to the manufacturer's protocol. In brief, we prepared DNA-reagent complexes at ratios of 1 : 2.5 (total volume = 500 *μ*L). Twenty minutes later, the complexes were added to each well. To investigate transfection efficiency, a green fluorescent protein (GFP) plasmid was introduced. 48  hours after transfection, flow cytometry analysis was conducted. The cells that displayed green fluorescence were examined through FL 1 channel (excitation: 488 nm, emission: 520 ± 10 nm; FACSCalibur, BD, USA). The efficiency was defined as the percentage of GFP-positive cells within all viable cells in 3 independent experiments. Hepatocytes treated without GFP served as control.

### 2.4. Reverse Transcription Polymerase Chain Reaction

Total RNA was extracted using TRIzol (Takara, Japan) from two-day cultured hepatocytes. RT-PCR was performed according to the manufacturer's instructions (PrimeScript RT-PCR Kit, Takara). The following gene-specific oligonucleotide primers were used for amplification: Pax6 (314 bp), CAG TCA CAG CGG AGT GAA TCA GC (forward) and GCC ATC TTG CGT AGG TTG CCC TG (reverse); Nkx6.1 (381 bp), GTT CCT CCT CCT CCT CTT CCT C (forward) and AAG ATC TGC TGT CCG GAA AAA G (reverse); and Isl1 (268 bp), GTG CGG AGT GTA ATC AGT ATT TGG (forward) and GTC ATC TCT ACC AGT TGC TCC TTC (reverse). Amplification conditions were initial denaturation at 94°C for 10 min, followed by 35 cycles of denaturation at 94°C for 30 sec, annealing at 60°C for 30 sec and extension at 72°C for 30 sec, and at last an extension step of 10 min at 72°C.

### 2.5. Real-Time Fluorescence Relative Quantitative PCR (qPCR)

Total RNA was obtained as above. The primers were the following: Gk (islet type), CAG AGA CAC AAC AAC CTT TTC CC (forward) and GCT GTC TCA CTG GCT GAC TT (reverse); Ins1, TTG GTG CAC TTC CTA CCC CT (forward) and CAC ACA CCA GGT AGA GAG CC (reverse); and Ins2, CCA TCA GCA AGC AGG AAG GTT A (forward) and CAG GTG GGA ACC ACA AAG GT (reverse). The SYBR-Green real-time PCR reaction solution was prepared and the PCR conditions were set according to the manufacturer's protocol (ABI, USA). The data were analyzed for target gene expression by the 2^−ΔΔCt^ method. Normal C57BL/6J mice islet was used as a control.

### 2.6. Western Blot Assay

Five days after transfection, proteins were isolated. The protein concentrations were determined by protein assay (KeyGEN, China). Electrophoresis on a 12% SDS polyacrylamide gel was performed and the protein was transferred to a PVDF membrane (Millipore, Germany). Protein bands were detected using an enhanced chemiluminescence system (Pierce Biotechnology, USA) with antibodies (Santa Cruz, USA).

### 2.7. Insulin and C-Peptide Detection

5-day cultured hepatocytes were treated with acid-ethanol at 4°C overnight. The supernatants were collected and the intracellular C-peptide levels were measured by an enzyme-linked immunosorbent assay (C-peptide-ELISA kit, Millipore). Insulin secretion was determined by an ELISA kit (Insulin-ELISA kit, Millipore) after exposure to Krebs-Ringer bicarbonate (KRB) buffer with 0.1% BSA containing various concentrations of glucose (0, 5, or 25 mM) as described [13]. The values of insulin and C-peptide were normalized relative to the total protein content which was detected by protein assay (KeyGEN, China).

### 2.8. Streptozotocin-Induced Diabetic Mice and Cell Transplantation

6-week-old male C57BL/6J mice (Laboratory Animal Center of Southern Medical University) were injected intraperitoneally with streptozotocin (STZ; Merck, Germany) at a dose of 180 mg/kg body weight. Diabetes was diagnosed by blood glucose levels >300 mg/dL (16.7 mM) on two consecutive measurements. Recombinant plasmids which encode Pdx1, Ngn3, and MafA (MNP) as well as null vectors (NV) were delivered into hepatocytes 3 days before transplantation. 6–8 × 10^6^ cells suspended in 0.2 mL PBS were transplanted into the liver parenchyma of diabetic mice through portal injection.

### 2.9. Glucose Tolerance Test

Mice were injected intraperitoneally with glucose at a dose of 1 g/kg body weight after 6 h fast. Blood samples were collected from the tail vein to monitor glucose levels at the indicated time points.

### 2.10. Statistical Analysis

All values are expressed as means ± SD. The differences were analyzed with two-sample Student's *t*-test, and *P* < 0.05 was considered to be significant.

## 3. Results

### 3.1. Transfection Efficiency in Our Experiment

**Figure 1:**
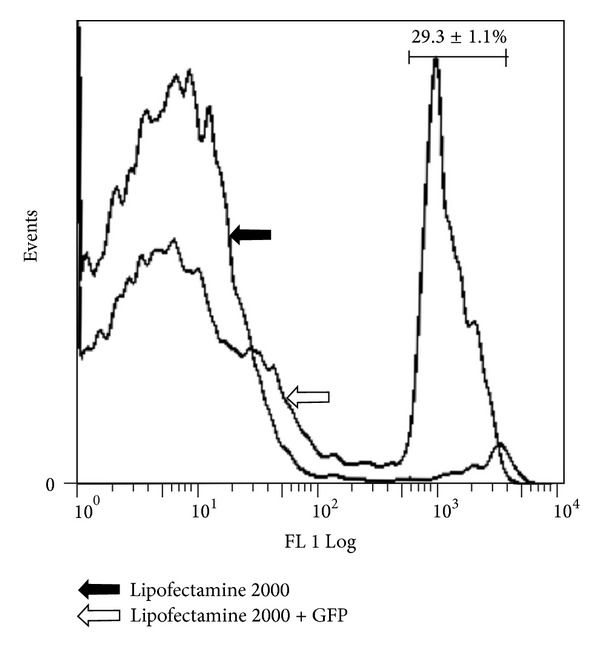
Flow cytometry quantitation of GFP-positive cells demonstrates that treatment with GFP results in a 16-fold higher number, compared with those without GFP; *n* = 3.

Transfection efficiency was recognized as the proportion of GFP-positive cells among all viable cells at 48 h after transfection. Our data indicated that transfection efficiency was 29.3 ± 1.1% (Figure 1), which was close to previous study [14].

### 3.2. Ectopic Coexpression of Pdx1, Ngn3, and MafA Markedly Promotes Hepatocytes-to-IPCs Reprogramming

**Figure 2:**
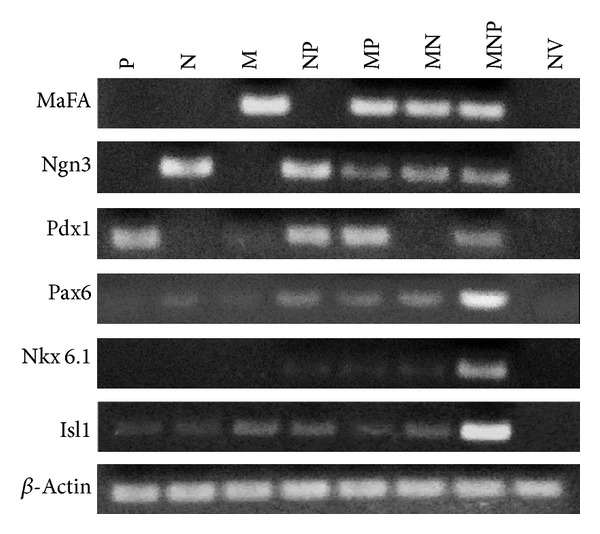
Gene expression profiles in transfected hepatocytes. Total RNA was isolated from hepatocytes treated with pPdx1 (P), pNgn3 (N), pMafA (M), pNgn3+Pdx1 (NP), pMafA+Pdx1 (MP), pMafA+Ngn3 (MN), pMafA+Ngn3+Pdx1 (MNP), and null vector (NV).

Using RT-PCR, we investigated the expression profiles of the transfected transcription factors and islet-related genes. As expected, the transfected transcription factor(s) was (were) detectable in corresponding group. Interestingly, some factors were found in their transfection-default group. For example, exogenous MafA could induce the expression of the endogenous Pdx1. On the other hand, the mRNA expression of pancreatic transcription factors including Pax6, Nkx6.1, and Isl1 in MNP group was higher than the other groups. However, the genes above were not detectable in control NV group (Figure 2).

**Figure 3:**
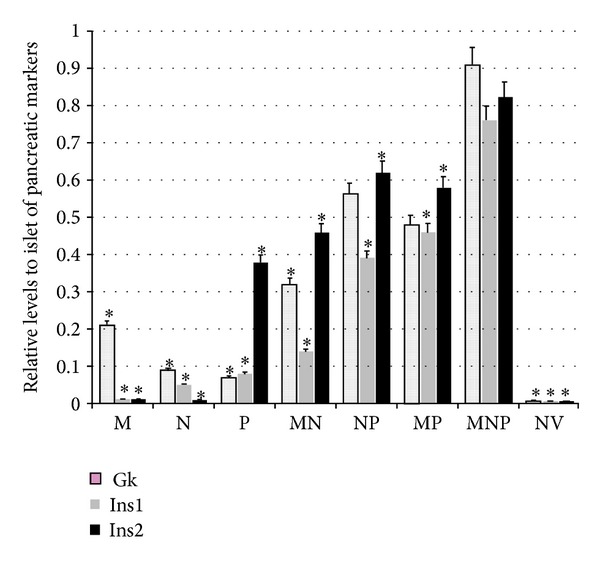
Relative levels to mice islet of endogenous pancreatic markers in 5-day cultured hepatocytes, evaluated by real-time PCR; *n* = 3. _ _*P < 0.05 versus MNP.

A better illustration for the extent of the transdifferentiation process is to quantify the expression of the endogenous pancreatic markers in transfected hepatocytes. Compared to MNP group, differences in the expression of islet-type Gk in bicistronic groups were not significant. It is documented that Ins2 is detectable in yolk sac and developing brain as well as in islet cells, while Ins1 appears in islet cells only. In these hepatocytes, the relative values of Ins2 and Ins1 in MNP group were 0.82 ± 0.04 and 0.76 ± 0.04, respectively, which were higher than that in the rest groups (Figure 3).

**Figure 4:**
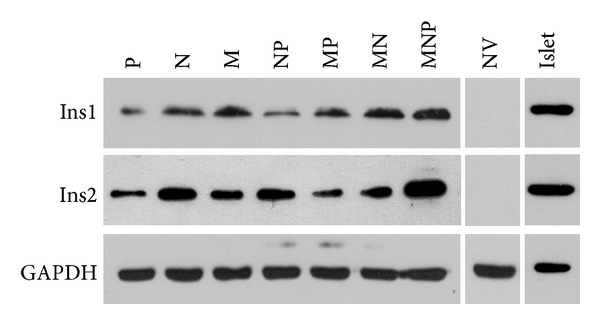
Protein levels of Ins1 and Ins2 in hepatocytes on day 5 after transfection, evaluated by Western blotting, with mice islet as positive control.

To confirm that transfected hepatocytes can express the target protein encodes by Ins1 and Ins2, we performed Western blot analysis. As shown in Figure 4, among all groups, level of both Ins1 and Ins2 protein was the highest in MNP group, except positive control group. These data suggested that transfection with Pdx1, Ngn3, and MafA activated an endocrine developmental shift in fully differentiated hepatocytes.

### 3.3. Triple Transfection Enables Hepatocytes to Reprogram into IPCs

**Figure 5:**
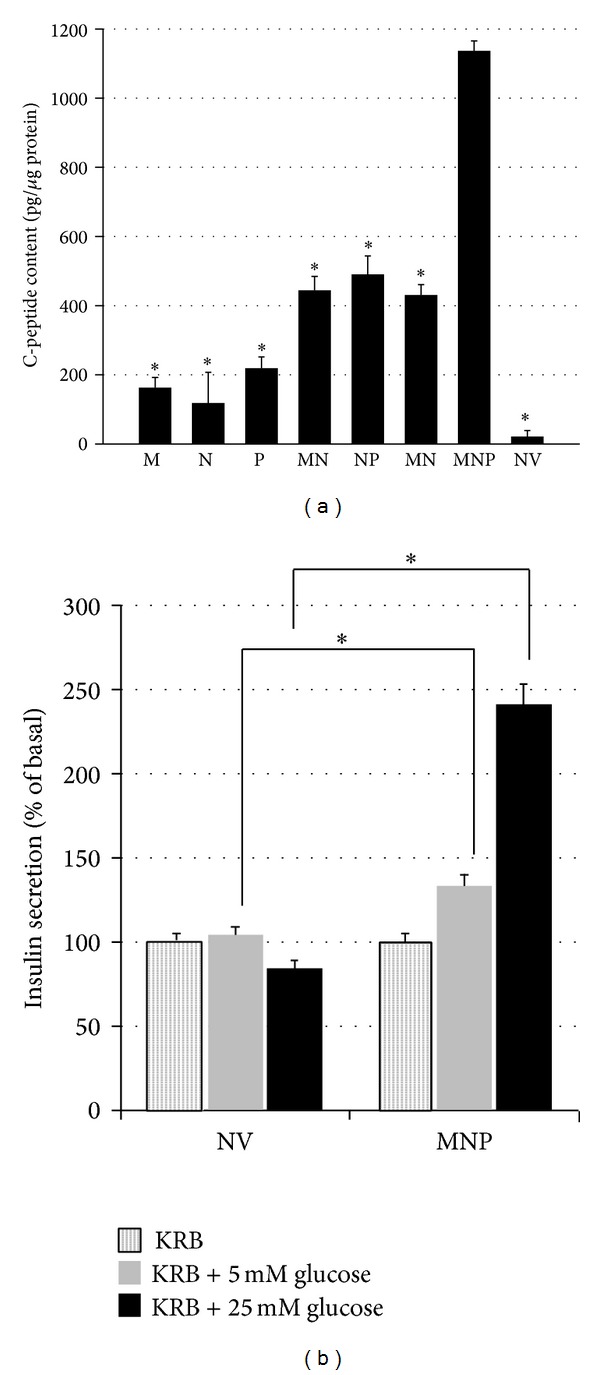
Evaluation of insulin synthesis and secretary ability in transfected cells. (a) Measurements of intracellular C-peptide were conducted by an ELISA kit; *n* = 3. (b) Glucose-induced insulin secretion from transfected hepatocytes. Values are mean ± SD of insulin in 3 different experiments, relative to 0 mM glucose. _ _*P < 0.05.

In order to examine insulin biosynthesizing in transfected cells, intracellular C-peptide contents were measured. The figure of C-peptide in MNP group which reached 1200 pg/*μ*g protein was significantly higher (2–120-fold) than the other groups (Figure 5(a)). Glucose-sensing ability and the coupling between glucose sensing and insulin secretion play an important role in pancreatic beta-cell function. Therefore, insulin secretion was evaluated after static incubation with glucose using a commercial ELISA kit. From Figure 5(b), we can see that the hormone was secreted in a glucose-responsive manner, in MNP group. These results together with data from C-peptide secretion implied that IPCs reprogrammed from hepatocytes with triple transfection closely resembles normal pancreatic beta-cells.

### 3.4. IPCs Ameliorate STZ-Induced Hyperglycemia In Vivo

**Figure 6:**
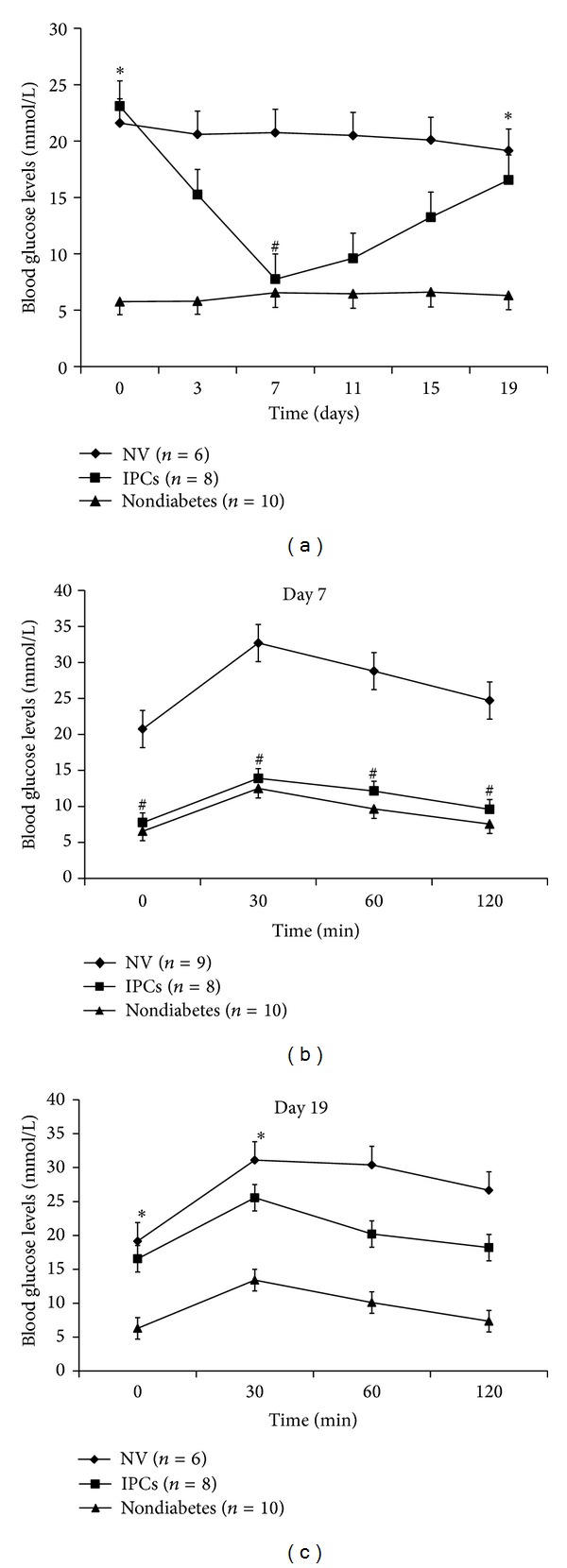
Effects on blood glucose levels by transplanting IPCs into diabetic mice at different times. A glucose tolerance test was performed, and glucose levels were determined from blood drawn from the tail vein. (a) Fasting blood glucose of mice until day 19. ((b) and (c)) Glucose levels during a glucose tolerance test of mice on days 7 and 19. Data are presented as mean ± SD. _ _*P > 0.05 versus NV; _ _^#^ *P* > 0.05 versus nondiabetes.

To further assess effectiveness of IPCs in maintaining glucose homeostasis, they were transplanted into diabetic mice, which were treated with STZ to specifically damage islet *β*-cells. Meanwhile, hepatocytes transfected with NV were transplanted as a negative control. A glucose tolerance test was conducted daily until day 19 after transplantation. As shown in Figure 6(a), 2 days after transplantation, fasting blood glucose levels were reduced by IPCs. Furthermore, IPCs-transplanted mice exhibited a complete reversal of hyperglycemia on day 7. It is noted that hypoglycemia was not observed (Figure 6(b)). These data suggested that transfection with Pdx1, Ngn3, and MafA multicistronic expression vector through Lipofectamine 2000 was a sufficient nonviral approach to trigger hepatocytes-to-IPCs reprogramming. However, profound effect of improving hyperglycemia was not obtained at day 19. Differences at the time point of 0 and 30 min during a glucose tolerance test between mice implanted with IPCs and NV-treated hepatocytes were not significant (Figure 6(c)).

## 4. Discussion

It has been confirmed that one kind of adult somatic cells can transdifferentiate into another one, without undergoing the process of dedifferentiation [9, 15, 16]. This was termed as cell direct reprogramming technology. It was demonstrated that the coexpression of Pdx1, Ngn3, and MafA in pancreatic exocrine cells was the most effective way for IPCs reprogramming [9]. In this study, we examined the potential of IPCs reprogramming by using multicistronic vectors-mediated coexpression of the three transcription factors in hepatocytes.

As promoters of hepatocytes-to-IPCs reprogramming, we focused on the three transcription factors: Pdx1, Ngn3, and MafA. Pdx1 is a “switch” of pancreas development and function, and Ngn3 is expressed in endocrine progenitors and is in charge of islet differentiation [17]. With respect to MafA, it is a key calibrator of glucose-responsive insulin secretion [18]. Despite that all the three factors could trigger the process, none of them could achieve the reprogramming alone, while the specific combination does. Similar results were obtained in our study. Substantial increase in insulin gene expression (Figures 3 and 4) and C-peptide (Figure 5(a)) was found in MNP group. Furthermore, IPCs responded to glucose challenge both in vitro (Figure 5(b)) and in vivo (Figure 6(b)). These results suggested that IPCs acquired the capacity of insulin synthesis, storing, secretion, and glucose-sensing. The exact mechanisms underlying the processes are still elusive at present. However, the following possibilities may be important in explaining it. Firstly, as mentioned above, embryological homology between hepatocytes and islet-cells may facilitate the transdifferentiation. Secondly, it could be attributed to the powerful synergistic effect of pancreatic transcriptional network including Pax6, Nkx6.1, and Isl1 which were elicited by triple overexpression promoting [19]. RT-PCR analysis revealed large induction of expression of Pax6, Nkx6.1, and Isl1 in MNP group (Figure 2). The powerful synergistic effect of pancreatic transcriptional network was proved to promote insulin expression [20, 21].

Besides embryological homology, there is another advantage when using hepatocytes as a source of IPCs. From Greek myth about Prometheus, ancient Greeks had realized the regenerative capacity of the liver. In modern times, it has been proved by many studies that injuries, including chemical insult and surgical remove, can trigger liver regeneration [22]. The regenerative procedure will finish within one week in rodents even there are only approximately 30% of liver left [23]. More importantly, the structure and function of the liver maintained during the repopulation [24, 25]. Additionally, laparoscopic approach has been considered as a safe and effective therapeutic option in hepatectomy. Thus, the acquirement of hepatocytes by laparoscopic approach makes autotransplantation possible, allowing diabetic patients to be the donors of themselves.

Viral vectors are attractive tools for gene delivery because of their high efficiency. Nevertheless, questions regarding toxicity, immunogenicity, and a probable risk for insertional mutagenesis following viral vectors are hurdles that may preclude their widespread use [26]. The success of gene therapy requires the development of the gene delivery vector. Owing to its ability to mediate stable transgene efficiency, large cloning capacity, devoid of eliciting a major humoral immune response, and lower cost, multicistronic vector has entered the realm of the current therapeutic gene transfer arena, recently [27]. By using it, we achieved the hepatocytes-to-IPCs reprogramming.

In conclusion, our study reveals that multicistronic vectors-mediated expression of Pdx1 together with Ngn3 and MafA in hepatocytes was a feasible regimen for inducing islet neogenesis in vitro and resulting in correction of diabetic state in vivo. Additional investigations are needed to prolong duration of euglycemia in animals. We believe that this information is valuable for cell replacement of diabetes.
